# Active Sulfate-Reducing Bacterial Community in the Camel Gut

**DOI:** 10.3390/microorganisms11020401

**Published:** 2023-02-04

**Authors:** Olga V. Karnachuk, Inna A. Panova, Vasilii L. Panov, Olga P. Ikkert, Vitaly V. Kadnikov, Igor I. Rusanov, Marat R. Avakyan, Lubov B. Glukhova, Anastasia P. Lukina, Anatolii V. Rakitin, Shahjahon Begmatov, Alexey V. Beletsky, Nikolai V. Pimenov, Nikolai V. Ravin

**Affiliations:** 1 Laboratory of Biochemistry and Molecular Biology, Tomsk State University, 634050 Tomsk, Russia; 2 Institute of Bioengineering, Research Center of Biotechnology of the Russian Academy of Sciences, Leninsky Prosp, bld. 33-2, 119071 Moscow, Russia; 3 Institute of Microbiology, Research Center of Biotechnology of the Russian Academy of Sciences, 119071 Moscow, Russia

**Keywords:** sulfate-reduction, camels, gut microbiota, *Desulfovibrio*, *Desulforamulus*, biogenic chalcopyrite

## Abstract

The diversity and activity of sulfate-reducing bacteria (SRB) in the camel gut remains largely unexplored. An abundant SRB community has been previously revealed in the feces of Bactrian camels (*Camelus bactrianus*). This study aims to combine the 16S rRNA gene profiling, sulfate reduction rate (SRR) measurement with a radioactive tracer, and targeted cultivation to shed light on SRB activity in the camel gut. Fresh feces of 55 domestic Bactrian camels grazing freely on semi-arid mountain pastures in the Kosh-Agach district of the Russian Altai area were analyzed. Feces were sampled in early winter at an ambient temperature of −15 °C, which prevented possible contamination. SRR values measured with a radioactive tracer in feces were relatively high and ranged from 0.018 to 0.168 nmol S cm^−3^ day^−1^. The 16S rRNA gene profiles revealed the presence of Gram-negative *Desulfovibrionaceae* and spore-forming *Desulfotomaculaceae*. Targeted isolation allowed us to obtain four pure culture isolates belonging to *Desulfovibrio* and *Desulforamulus*. An active SRB community may affect the iron and copper availability in the camel intestine due to metal ions precipitation in the form of sparingly soluble sulfides. The copper-iron sulfide, chalcopyrite (CuFeS_2_), was detected by X-ray diffraction in 36 out of 55 analyzed camel feces. In semi-arid areas, gypsum, like other evaporite sulfates, can be used as a solid-phase electron acceptor for sulfate reduction in the camel gastrointestinal tract.

## 1. Introduction

Sulfate-reducing bacteria (SRB) are a common constituent of the gut microbiota in humans and other animals [1,2,3,4,5,6,7,8]. SRB activity in the human gastrointestinal tract has been associated with different pathologies including inflammation, ulcerative colitis, and colorectal cancer [3,9,10,11,12,13]. On the other hand, hydrogen sulfide produced by *Desulfovibrio* in the gut has been shown to improve insulin secretion and sensitivity [14] and provide fixed nitrogen [15]. *Desulfovibrio* has been reported to be the dominant SRB genus in the human intestine [16]. *Desulfovibrio* spp. were the predominant SRB in the piglet gut [17] and have been documented in the ruminal content of cows, sheep, reindeer, and red deer [7]. H_2_-uptake hydrogenases have been found in 47 *Desulfovibrio* composite genomes (MAGs) from dairy cattle (*Bos taurus*), water buffalo (*Bubalus bubalis*), yak (*Bos grunniens*), goat (*Capra aegagrus*), sheep (*Ovis aries*), roe deer (*Capreolus pygargus*), and water deer (*Hydropotes inermis*) [18]. *Desulfovibrio* was among the 10 most common genera from the cecum in horses [19]. The diversity and activity of SRB in the camel gut remains largely unexplored. Ming and co-authors revealed an abundant sulfate-reducing community, mainly *Desulfovibrio*, in fecal samples from Mongolian domestic Bactrian camels as well as Mongolian wild Bactrian camels [20]. The abundance of *Desulfovibrio* in the gastrointestinal tract of camels is attributed by the authors to their bioremediation potential, including the precipitation of toxic metals in the form of sulfides, which helps camels to survive in harsh environments and feed on poisonous plants.

Biogenic iron sulfides, such as pyrite (FeS_2_) and others, are considered as geochemical markers of SRB activity in various biotopes. A distinctive feature of hydrogen sulfide, the end product of microbial sulfate reduction, is its high reactivity leading to the formation of sparingly soluble metal sulfides. The role of biogenic H_2_S in the formation of digenetic pyrite (FeS_2_) and other iron sulfides in environmental biotopes is well recognized [21]. The formation of crystalline iron sulfides as a result of SRB activity in the intestine has received less attention compared to natural environments. Low-soluble greigite and pyrite formation by *Desulfovibrio desulfuricans* AY5 isolated from a fecal sample of a person with autistic spectrum disorders has been demonstrated [22]. No crystalline phases or Cu sulfides were detected in the batch culture of *Tissierella* sp. P1, an intestinal bacterium that produces H_2_S from peptone [23]. In our preliminary study of the mineralogical composition of camel feces, we detected a copper iron sulfide, chalcopyrite (CuFeS_2_). We hypothesized that the CuFeS_2_ occurrence may indicate an active process of microbial sulfate reduction in the camel gut. This study aims to test the hypothesis by combining the 16S rRNA gene profiling, sulfate reduction rate measurement with a radioactive tracer, and SRB targeted isolation from camel fecal samples.

## 2. Materials and Methods

### 2.1. Sampling and Mineralogical Characteristics of Camel Feces

Fecal samples were collected from domestic camels continuously grazing freely wild vegetation, including thorny shrubs, in the Chagan River valley close to the Beltyr village. The site is located in the south-eastern part of the Russian Altai at the elevation at 1959 m above sea level in the permafrost area. The Bactrian two-humped camels (*Camelus bactrianus*) inhabit the cold deserts of southern areas of central (Kazakhstan, Iran) and eastern (Russia, Mongolia, China) Asia [24]. Bactrian camels have been bred in the south-east of the Altai Mountain, on the Russian border with Mongolia, since the time of the Silk Road. Domestic camels are grazing freely on natural grasslands and shrublands in semi-arid steppe and arid mountain steppe all year round. Samples were collected from 55 individual healthy adult animals directly after defecation on 18 November 2021. The ambient air temperature at the time of sampling was −15 °C, which reduced the possibility of contamination by microorganisms from soil and air. The samples were collected aseptically into sterile plastic bags. The fresh fecal samples were split into two parts and transported to the laboratory, where the part for DNA isolation was stored at −80 °C and the part for SRB cultivation was kept in a refrigerator. A sample of saline soil, where camels come to lick the salt, was also collected in a sterile plastic bag for mineralogical analysis.

The fecal and soil samples were air-dried and ground manually. Powder XRD was performed with a Rigaku Ultima 4 diffractometer (Rigaku Corp., Tokyo, Japan) with CuKα radiation. The samples were packed into zero-background quartz sample holders and step-scanned at the 2θ range from 10° to 75° using a 2θ step interval of 0.02° and a counting time of 0.8 s. The diffraction patterns were analyzed with the Crystallographica-Search Match software and the PDF-4 database (International Centre for Diffraction Data, http://www.icdd.com accessed on 31 October 2022).

### 2.2. Measurement of Sulfate-Reduction Rate with Radioactive Tracer

Radioactive sulfate was used to determine the sulfate-reduction rates (SRR) in camel feces. Feces were placed in sterile 5 mL syringes sealed with butyl rubber stoppers, which received aliquots (300 µL) of Na_2_^35^SO_4_ (3 µCi ‘Perkin-Elmer’, Waltham, MA, USA) by injection through the butyl rubber stopper. The syringes were incubated in the dark at 37 °C, for 24 h followed by the addition of 1 mL of 2M KOH to terminate the reaction and fix sulfide. Radioactivity was measured in the acid volatile sulfide (AVS), H_2_S and FeS, and chromium-reducible sulfur (CRS) fractions, which included pyrite, and elemental and organic sulfur, as previously described [25,26]. Sulfate concentration was analyzed by ion chromatography (Dionex). The average SRR and standard deviation were calculated from triplicate incubations.

### 2.3. SRB Enrichments and Pure Culture Isolation

The initial enrichments were set up immediately upon fecal samples arrival to the lab in freshwater Widdel and Bak (WB) medium [27] that contained (per liter) 4 g Na_2_SO_4_, 0.2 g KH_2_PO_4_, 0.25 g NH_4_Cl, 1 g NaCl, 0.4 g MgCl_2_·6H_2_O, 0.5 g KCl, 0.113 g CaCl_2_, 2 mL of vitamin solution, 1 mL of microelement solution, 1 mL each of Na_2_SeO_3_ (final concentration 23.6 µM), and Na_2_WO_4_ (24.2 µM) solutions, and solidified with 1.5% agar. Medium was adjusted to pH 7.2 with NaHCO_3,_ and Na_2_S·9H_2_O was used as a reducing agent. Each cultivation vial received a Fe^0^ wire as previously described [28]. Formate (7.5 mM) and acetate (2 mM) was used as an electron donor and carbon source, respectively, to isolate the pure culture initially. Lactate (18 mM) was used for the subsequent cultivations. The enrichment cultures were incubated at 37 °C. The 16S rRNA genes were amplified using the primer pair 27F and 1492R and sequenced commercially by Syntol Co. (Moscow, Russia) using the Sanger method.

### 2.4. 16S rRNA–Based Microbial Community Profiling

Total genomic DNA from camel feces was extracted using a Power Soil DNA isolation kit (MO BIO Laboratories, Inc., Carlsbad, CA, USA) and stored at −20 °C. The 16S rRNA gene fragments were amplified by PCR using the universal primers 341F (5′-CCTAYGGGDBGCWSCAG-3′) and 806R (5′-GGACTACNVGGGTHTCTAAT-3′). PCR fragments were barcoded using the Nextera XT Index Kit v.2 (Illumina, San Diego, CA, USA) and sequenced on the Illumina MiSeq (2 × 300 nt paired-end reads). Overlapping reads were merged using FLASH v.1.2.11 [29]. Low-quality reads were excluded, and the remaining sequences were clustered into operational taxonomic units (OTUs) at 97% identity using the Usearch program [30]. Chimeric sequences were removed during clustering by the Usearch algorithm. To calculate the relative abundances of OTU, all 16S rRNA gene sequences were mapped to OTU sequences at 97% global identity threshold by Usearch. OTUs comprising only a single read were discarded. The taxonomic identification of OTUs was performed by searches against the SILVA v.138 rRNA sequence database using the VSEARCH v. 2.14.1 algorithm [31]. The alpha diversity indices at a 97% OTU cut-off level were calculated using Usearch. To avoid sequencing depth bias, the number of reads generated for each sample were randomly sub-sampled to the size of the smallest set (KV116 sample) using the QIIME 2 2022.8 tool [32].

## 3. Results

### 3.1. Mineralogical Composition of the Camel Feces and Soil Sample

**Figure 1:**
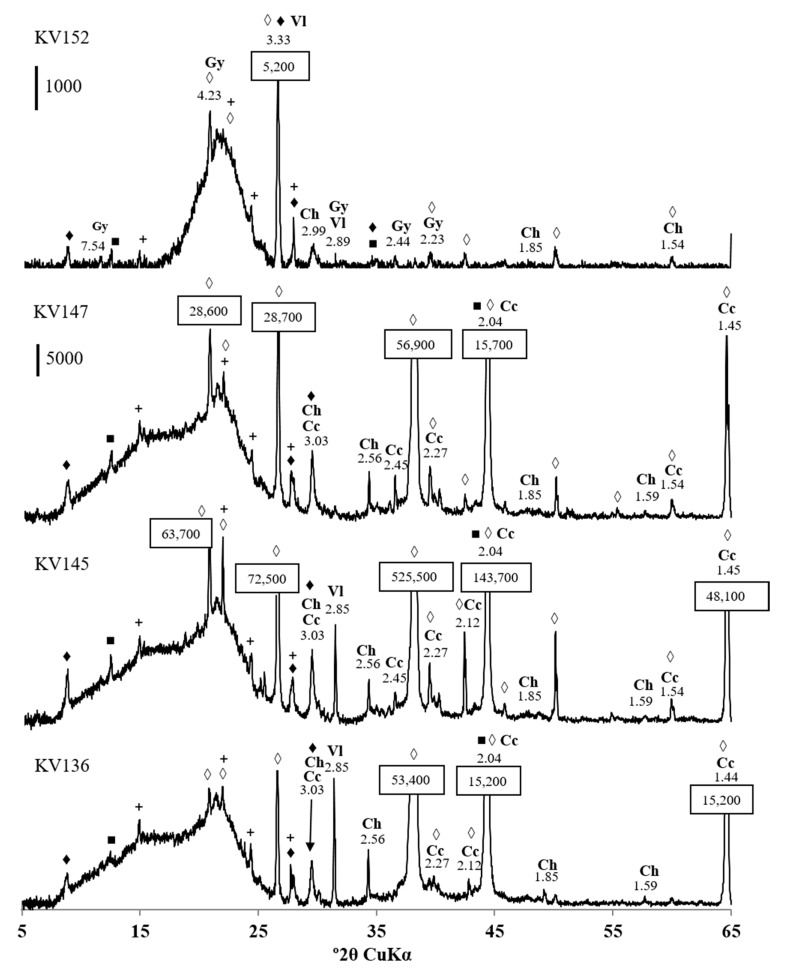
X-ray diffraction patterns of fecal samples KV152, KV147, KV145, and KV136. Letter codes: Ch = chalcopyrite, CuFeS_2_; Vl = villamaninite, CuS_2_; Gy = gypsum, CaSO_4_; Cc = calcite, CaCO_3_. The diagnostic peaks for muscovite (♦), clinochlore (■), quartz (◊), and albite (+) are indicated. The vertical bar shows the scale of relative counts.

The mineralogical composition of the camel feces revealed the presence of iron copper and copper sulfides, including chalcopyrite (CuFeS_2_) and villamaninite (CuS_2_) (Figure 1). Villamininte is a rare copper sulfide with small amounts of other elements [33]. In total, 36 out of 55 analyzed camel fecal samples contained chalcopyrite, but only three showed diagnostic peaks for villamaninite. The feces also contained alumosilicates: quartz (SiO_2_), albite (NaAlSi_3_O_8_), muscovite (K,Na)Al_2_(Si,Al)_4_O_10_(OH)_2_), and others. Gypsum (CaSO_4_·2H_2_O) was present in 35 samples and calcite, CaCO_3_, in 27 samples.

**Figure 2:**
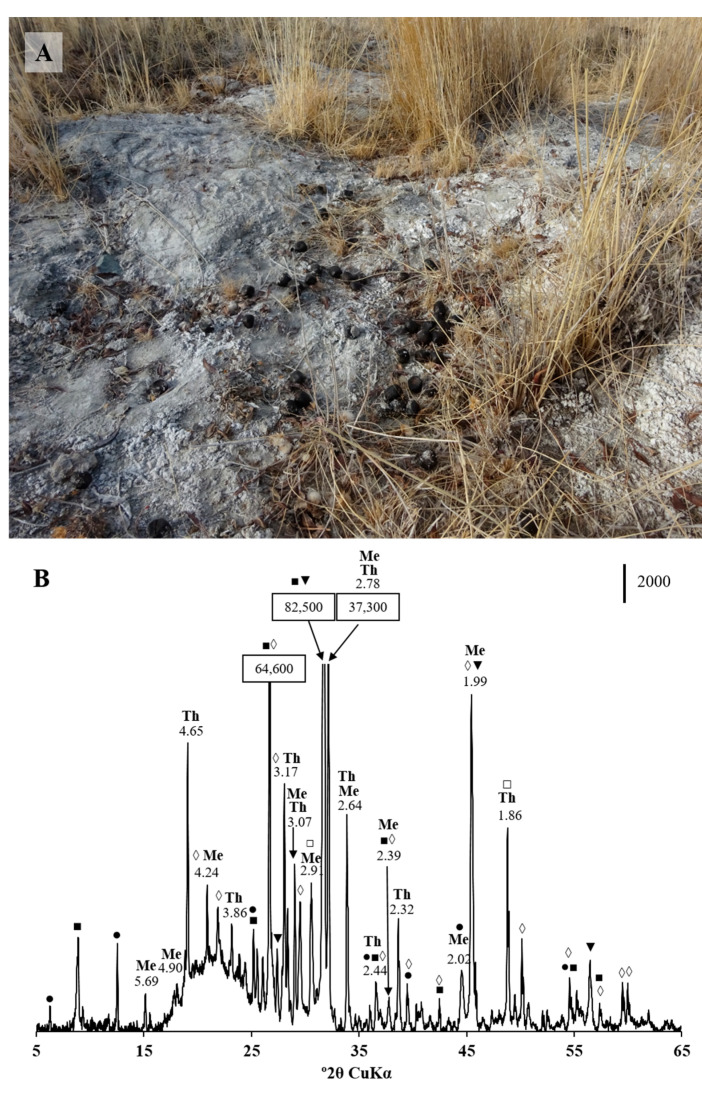
(**A**) Saline soil with camel feces, sampled for XRD analysis, and (**B**) X-ray diffraction pattern of the soil sample. Letter codes: Th = thenardite, Na_2_SO_4_; Me = melanterite, Fe^+2^SO_4_·7H_2_O. The diagnostic peaks for muscovite (■), clinochlore (●), calcite (□), quartz (◊), and halite (▼) are indicated. The vertical bar shows the scale of relative counts.

The mineralogical composition of the saline soil revealed the presence of an anhydrous sodium sulfate, thenardite (Na_2_SO_4_), and hydrous iron sulfate, melanterite (Fe_2_SO_4_·7 H_2_O) (Figure 2). Halite (NaCl) also occurs in the saline soils.

### 3.2. Composition of Fecal Microbiomes

**Figure 3:**
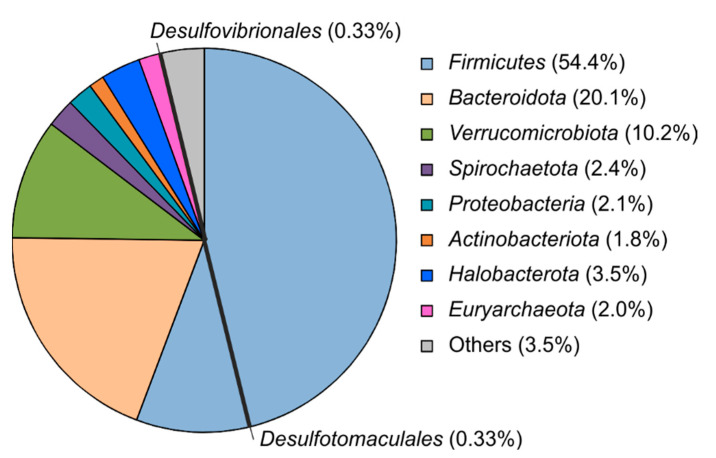
Microbial communities of camel feces at the phylum level and sulfate-reducing lineages (*Desulfotomaculales* and *Desulfovibrionales*). Relative abundances (% of the total 16S rRNA gene sequences, average of 55 samples) are shown after taxon names.

To characterize the taxonomic compositions of microbial communities a total of 868,359 sequences of 16S rRNA gene fragments were determined for 55 analyzed fecal samples and clustered into 6167 OTUs at the level of 97% sequence identity. The number of species-level OTUs present in individual samples ranged from 580 to 1282 (Supplementary Table S1). The results of the taxonomic classification of the OTUs are shown in Figure 3 and in Supplementary Table S2. The fecal microbiomes of camels were dominated by the phyla *Firmicutes* (from 39.7% to 70.2% of all 16S rRNA gene sequences, on average 54.4%), *Bacteroidota* (from 8.4% to 29.3%, on average 20.1%). Among the *Firmicutes*, the most numerous groups were *Oscillospiraceae*, *Lachnospiraceae, Christensenellaceae*, *Ruminococcaceae*, *Monoglobaceae*, *Peptostreptococcaceae*, *Anaerovoracaceae*, and uncultured family-level lineages UCG-010, UCG-014, and ‘Eubacterium coprostanoligenes group’, as defined in the SILVA taxonomy. Most of the *Bacteroidetes* were assigned to the families *Rikenellaceae*, *Bacteroidaceae*, *Prevotellaceae*, and uncultured lineages ‘M2PB4-65 termite group’, p-251-o5, F082, and UCG-001 of the order *Bacteroidales*. Other abundant bacterial phyla were *Verrucomicrobiota* (on average 10.2%, mostly *Akkermansia* sp. and the candidate genus WCHB1-41), *Spirochaetota* (2.4%, mostly *Treponema* sp.), *Proteobacteria* (2.1%), and *Actinobacteriota* (1.8%). Archaea were mostly represented by methanogens of the phyla *Halobacterota* (3.5%) and *Euryarchaeota* (2.0%); the most numerous OTUs were assigned to the genera *Methanocorpusculum* and *Methanobrevibacter*. Other microbial phyla accounted on average for less than 1% of 16S rRNA gene reads.

Among lineages known to comprise sulfate-reducing microorganisms, the orders *Desulfotomaculales* (*Firmicutes*) and *Desulfovibrionales* (*Desulfobacterota*) were identified, each accounting for about 0.3% of the 16S rRNA gene sequences. Most of the sequences assigned to the *Desulfotomaculales* belonged to two OTUs, comprising on average 0.26% and 0.05% of the community, and phylogenetically distant from known species. Considering cultured isolates, the closest relative of these OTUs was *Desulfoscipio (Desulfotomaculum) geothermicum* strain B2T but the level of 16S rRNA gene sequence identity was only 92.3% and 90.1%. One OTU was assigned to the genus *Desulfofundulus,* but it accounted on average for 0.01% of the microbiomes and was detected in only a few animals.

Most of the sequences assigned to *Desulfovibrionales* were phylogenetically close to the genera ‘*Mailhella*’ (0.23%) and *Desulfovibrio* (0.09%) of the family *Desulfovibrionaceae*. Particularly, the most numerous OTU (0.22%) showed 93.1% sequence identity with ‘*Mailhella*’ sp., a sulfate-reducing bacterium from the cecum of laying hens [34]. Several OTUs represented the genus *Desulfovibrio*; the most abundant of them (0.06%) showed 94.9% sequence identity to *Desulfovibrio desulfuricans* and probably represented a distinct species of this genus.

### 3.3. Sulfate Reduction Rate

**Figure 4:**
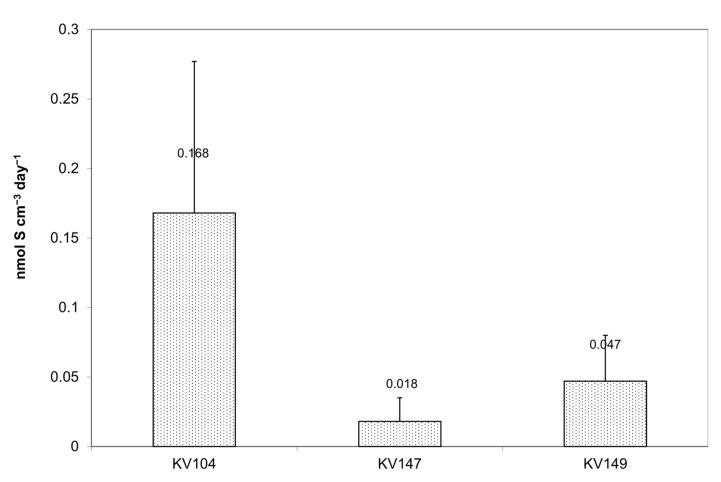
The sulfate reduction rate (SRR) measured in samples KV104, KV147, and KV 149. The vertical bars show the standard deviation.

The sulfate reduction rate (SRR) measured in three fecal samples was relatively high and varied from 0.018 to 0.168 nmol S cm^−3^ day^−1^ (Figure 4). The acid volatile sulfide fraction (AVS), which includes H_2_S and FeS, was the only product of ^35^SO_4_^2−^ reduction in samples KV147 and KV149. No tracer was detected in CRS, which may include pyrite and elemental and organic sulfur. On the contrary, the CRS fraction reached up to 30% in sample KV104, implying that metal sulfides may be formed even within 24 h of incubation.

### 3.4. SRB Cultivation

Since the 16S rRNA gene profiling revealed the occurrence of SRB belonging to *Desulfovibrionales* and *Desulfotomaculales* in camel feces, the targeted isolation has been applied to members of these lineages. The initial SRB enrichment cultures were set up with a mixture of formate (7.5 mM) and acetate (2 mM) as an electron donor to prevent overgrowth with the abundant heterotrophic bacteria on the medium with organic acids [35]. The incubation temperature was 37 °C. The electron donor was changed to lactate (18 mM) after the sulfidogenic growth appearance. Single colony isolation followed by multiple serial dilutions on the WB medium with lactate as an electron donor allowed us to obtain three pure culture isolates, designated as strain 1211, strain 1214, and strain 1223. Additional enrichment culture exposure to elevated temperature conditions at 90 °C for 20 min allowed us to isolate a spore-forming sulfidogen, designated strain 1198.

**Figure 5:**
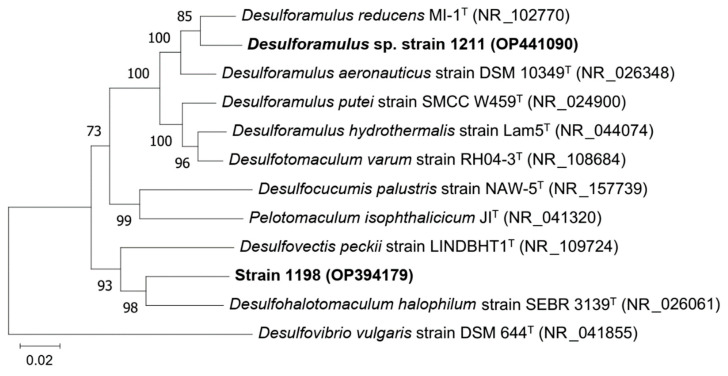
16S rRNA gene-based neighbor-joining tree showing the phylogenetic position of strains 1211 and 1198. The percentage of replicate trees in which the associated taxa clustered together in the bootstrap test (1000 replicates) are shown next to the branches. The evolutionary distances were computed using the maximum composite likelihood method and are in the units of the number of base substitutions per site. All ambiguous positions were removed for each sequence pair (pairwise deletion option). There were a total of 1658 positions in the final dataset. Evolutionary analyses were conducted in MEGA11.

The 16S rRNA gene sequence of strain 1211 placed it within the genus *Desulforamulus* (*Desulfotomaculales*) (Figure 4). The closest relatives of the strain were *D. reducens* with sequence similarity of 97.1% and *D. aeronauticus* (96.1%). Considering the 16S rRNA gene sequence similarity boundary cutoff of 98.7% [36], strain 1211 may represent a novel species of the genus *Desulforamulus*. Strain 1211 could grow at 4% of NaCl in the medium. A spore-forming strain 1198 was phylogenetically distant from known SRB (Figure 5). The closest relative of the strain 1198 was *Desulfohalotomaculum halophilum* with a 16S rRNA gene sequence similarity of 92.0%.

**Figure 6:**
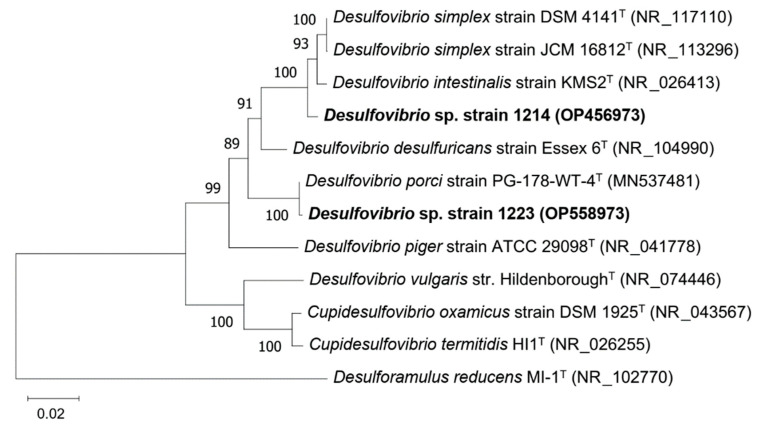
16S rRNA gene-based neighbor-joining tree showing the phylogenetic position of strains 1211 and 1198. The percentage of replicate trees in which the associated taxa clustered together in the bootstrap test (1000 replicates) are shown next to the branches. The evolutionary distances were computed using the maximum composite likelihood method and are in the units of the number of base substitutions per site. All ambiguous positions were removed for each sequence pair (pairwise deletion option). There were a total of 1590 positions in the final dataset. Evolutionary analyses were conducted in MEGA11.

Phylogenetic analyses placed strains 1214 and 1223 within the order *Desulfovibrionales*. Strain 1214 was a close relative of *Desulfovibrio simplex* with the 16S rRNA gene similarity of 98.9% (Figure 6). Unlike *D. simplex* [37], strain 1214 used glucose, fructose, and sucrose as electron donors for sulfate reduction, and could grow at 3% of NaCl in the medium. The 16S rRNA gene sequence of strain 1223 was 99.9% similar to that of *Desulfovibrio porci* (Figure 6), assuming that strain 1223 is a novel strain of *D. porci* isolated recently from swine pig feces under a large cultivation project, called the ‘Pig intestinal bacterial collection’ [38].

## 4. Discussion

The fecal microbiomes of camels were dominated by the phylum *Firmicutes* followed by *Bacteroidota* and *Verrucomoicrobiota*. A similar diversity pattern was described previously for Bactrian camels from Gobi-Altai region of Mongolia and from Inner Mongolia, China [20]. Our results on the SRR measurements and 16S rRNA gene profiling of fecal samples demonstrate that active dissimilatory sulfate reduction occurs in the camel intestine. Thus far, *Desulfovibrio* was considered the dominant SRB genus in the intestines of humans and other animals [3,7,16,17,18,19]. Particularly, *Desulfovibrio* was abundant in the fecal microbial communities of Mongolian wild and domestic Bactrian camels [20]. Therefore, the detection of *Desulfovibrio* in the microbial communities of camel feces was an expected result. In addition, molecular analysis and cultivation revealed spore-forming sulfidogenic *Firmicutes* inhabiting the camel intestine. Given the 92.0% similarity of the 16S rRNA gene to its closest relative, *D. halophilum*, strain 1198 may represent a novel genus within *Desulfotomaculaceae*. Given the similarity of the 16S rRNA gene to the closest relative of *D. reducens*, strain 1211 may represent a new species of the genus *Desulforamulus*. The first SRB pure culture isolated from animals was *Desulforamulus ruminis* (formerly *Desulfotomaculum ruminis*), a relative of strain 1211. *D. ruminis* was isolated by G. S. Coleman in the 1950s from the rumen of hay-fed sheep [9]. The large amount of toxic sulfide in ruminants has been a concern due to the presence of sulfate in grass and hay [39]. More recent genome sequencing revealed in *D. ruminis* a taurine degradation pathway, an organic compound that is widely distributed in animal tissues, especially the large intestine, and can provide an electron acceptor (sulfite) in biotopes depleted of sulfate [40].

16S rRNA gene profiling revealed that a significant share of *Desulfovibrionaceae* from camel feces was a relative of ‘*Mailhella*’ sp. The genus ‘*Mailhella*’ and its cultivated members have not yet been validly published. The first cultivated bacterium belonging to this genus was isolated from a fresh stool sample from a healthy French patient and was named ‘*Mailhella massiliensis*’ [41]. The type strain of the species has been poorly characterized and no genomes of the genus are available. Recently, ‘*Mailhella*’ was detected in the cecum of laying hens using 16S rRNA sequencing [34], confirming its role in H_2_S production in animals.

The relatively high abundance of *Spirochaetota* revealed in the studied camel fecal samples may be an indirect consequence of a significant H_2_S amount produced by sulfate-reducers. Spirochetes are characterized by a lack of advanced mechanisms for oxygen stress defense and often co-exist with sulfidogenes producing H_2_S, a strong reductant, in the environmental biotopes [42]. In general, the composition of camel feces microbiome revealed in our study corroborates the previously reported dominance of *Firmicutes* and *Verrucomicrobia* in Inner Mongolian domestic and wild Bactrian camels [23].

Hydrogen sulfide produced by SRB binds iron and other metals in the form of low-soluble sulfides. The formation of biogenic crystalline iron sulfides–pyrite, marcasite, greigite, and mackinawite by SRB is well documented [20,43,44,45,46]. Various copper sulfides, including covellite, chalcocite, and chalcopyrite, have been detected in SRB pure cultures [47,48,49]. Despite the solid recognition of SRB as an intestine inhabitant, little attention was paid to the metal precipitation by biogenic H_2_S produced by sulfidogenic bacteria in this environment. Low-soluble metal formation in the gastrointestinal tract can have two consequences. First, it reduces the bioavailability of metals. A previous study demonstrated the formation of insoluble greigite and pyrite by *Desulfovibrio desulfuricans* AY5 isolated from a person with autistic spectrum disorders [21]. Iron and copper deficiencies have been documented for this neurodevelopmental disease [50]. The formation of copper iron sulfide, chalcopyrite (CuFeS_2_), is an overlooked consequence of active sulfate reduction in the intestine. The formation of insoluble sulfides implies that copper can precipitate with iron and be excreted from the organism as chalcopyrite. Chalcopyrite as a diagenetic mineral requires the preliminary formation of iron sulfides, and its formation reaction proceeds through a series of metastable Cu-Fe-sulfide intermediaries [51]. Periods of undernutrition for copper and zinc have been reported for camel metabolism [52]. Nutritional factors are believed to control copper status in camel rather than physiology [53]. The authors report a significant effect of copper supplements in the form of copper sulfate salt. Iron deficiency has not been reported in animals grazing in natural conditions [53]. The formation and excretion of chalcopyrite due to active sulfate reduction in the camel gut can be an overlooked cause of iron and copper deficiency.

On the other hand, the precipitation of insoluble metal sulfides in the intestine can detoxify harmful metal ions. The significant share of *Desulfovibrio* in the camel intestine observed in a previous study was determined to aid the camel’s survival in harsh conditions and enable them to consume a diet of sharp, thorny and poisonous plants in a semi-arid environment [20]. The genome analysis of *D. porci* revealed genes coding for the HydH/G zinc/lead two-component system, suggesting resistance to high environmental zinc and lead levels [38].

It is plausible that in arid and semi-arid environments, the camel food can be enriched with sulfate, an electron acceptor for SRB. Evaporate gypsum is a common mineral in the desert environments due to climatic conditions, and in some locations, it even crystallizes as desert roses. The appearance of gypsum nodules in sedimentary rocks of the Oligocene-Lower Miocene Kosh-Agach Formation was reported [54]. Camels require salt supplements in their food and often graze on pastures with salty plants and bushes [55]. A mineralogical analysis of saline soil located near a pasture area where camels come to lick salt, did not reveal gypsum presence. However, two other sulfates, thenardite (Na_2_SO_4_) and melanterite (Fe_2_SO_4_·7H_2_O), were discovered in the soil sample. Thenardite is a common mineral for arid evaporite environments. X-ray diffraction (XRD) analysis revealed gypsum presence in 63.6% of the studied camel feces, its source remains unresolved. CaSO_4_ can serve as a solid-phase electron acceptor for SRB. Sulfide formation by *Desulfovibrio* spp. from gypsum has been shown to be almost compatible in rate and quantity to that produced from soluble sulfate [56]. The gypsum used in animal bedding has been shown to be a source of H_2_S, produced by *Desulfovibrio* in swine finishing facility waste [35]. Use of the sulfate entity from gypsum by SRB can result in the formation of calcium carbonate, which is proved to be produced as a result of dissimilatory sulfate reduction [57,58]. CaCO_3_ was present in 49.1% of the studied camel feces.

In conclusion, an active sulfate-reducing consortium is present in the Bactrian camel intestine freely grazing on semi-arid mountain pastures in the Kosh-Agach district of the Russian Altai area. Metal sulfides, chalcopyrite, and villamaninte, detected in camel feces can be considered as geochemical markers of microbial sulfate reduction. Iron copper sulfide, chalcopyrite (CuFeS_2_) was observed in 65% of the studied camel feces. Both *Desulfovibrionales* and *Desulfotomaculales* were present in the SRB consortium observed in camel feces. Evaporate gypsum intake may support dissimilatory sulfate reduction in the camels’ gut and input into copper and iron binding into low-soluble sulfides and excretion from the organism.

## Data Availability

The raw data generated from 16S rRNA gene sequencing have been deposited in the NCBI Sequence Read Archive (SRA) and are available under the accession numbers SRR17182714-SRR17182718, SRR17182624-SRR17182633, SRR17182635-SRR17182644, SRR17182646-SRR17182666, and SRR17182668-SRR17182676. The GenBank accession number for the 16S rRNA gene sequences of *Desulforamulus* sp. strain 1211, *Desulfovibrio* sp. strain 1214, *Desulfovibrio porci* strain 1223, and strain 1198 are OP441090, OP456073, OP558973, and OP394179, respectively.

## Supplementary Materials

The following supporting information can be downloaded at: https://www.mdpi.com/article/10.3390/microorganisms11020401/s1. Table S1: Sequencing statistics and diversity indices; Table S2: Relative abundance and taxonomic classification of OTUs.
